# HIPK2 C-terminal domain inhibits NF-**κ**B signaling and renal inflammation in kidney injury

**DOI:** 10.1172/jci.insight.175153

**Published:** 2024-03-21

**Authors:** Ye Feng, Zhengzhe Li, Heather Wang, Bi-Cheng Liu, Kyung Lee, John Cijiang He

**Affiliations:** 1 Department of Medicine/Nephrology, Icahn School of Medicine at Mount Sinai, New York, New York, USA.; 2 Institute of Nephrology, Zhong Da Hospital, Southeast University School of Medicine, Nanjing, Jiangsu, China.; 3 Renal Section, James J. Peters Veterans Affairs Medical Center, New York, New York, USA.

**Keywords:** Nephrology, Fibrosis, NF-kappaB

## Abstract

HIPK2 is a multifunctional kinase that acts as a key pathogenic mediator of chronic kidney disease and fibrosis. It acts as a central effector of multiple signaling pathways implicated in kidney injury, such as TGF-β/Smad3-mediated extracellular matrix accumulation, NF-κB–mediated inflammation, and p53-mediated apoptosis. Thus, a better understanding of the specific HIPK2 regions necessary for distinct downstream pathway activation is critical for optimal drug development for CKD. Our study now shows that caspase-6–mediated removal of the C-terminal region of HIPK2 (HIPK2-CT) lead to hyperactive p65 NF-κB transcriptional response in kidney cells. In contrast, the expression of cleaved HIPK2-CT fragment could restrain the NF-κB transcriptional activity by cytoplasmic sequestration of p65 and the attenuation of IκBα degradation. Therefore, we examined whether HIPK2-CT expression can be exploited to restrain renal inflammation in vivo. The induction of HIPK2-CT overexpression in kidney tubular cells attenuated p65 nuclear translocation, expression of inflammatory cytokines, and macrophage infiltration in the kidneys of mice with unilateral ureteral obstruction and LPS-induced acute kidney injury. Collectively, our findings indicate that the HIPK2-CT is involved in the regulation of nuclear NF-κB transcriptional activity and that HIPK2-CT or its analogs could be further exploited as potential antiinflammatory agents to treat kidney disease.

## Abstract 2

The HIPK2 c-terminal domain is anti-inflammatory and attenuates acute kidney injury in murine models.

## Graphical abstract

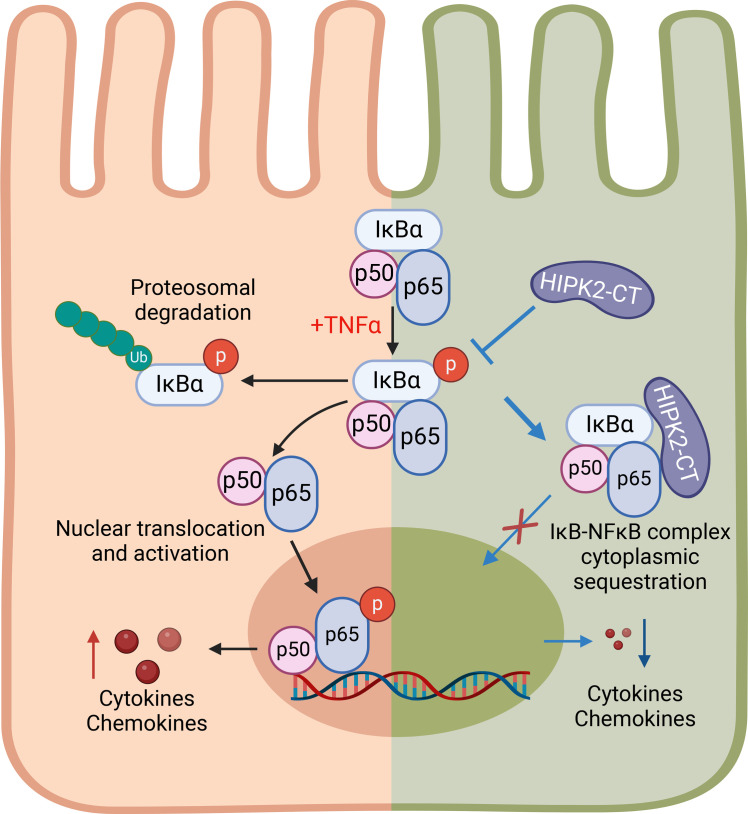

## Introduction

Renal inflammation and fibrosis are intricately linked pathological processes that mediate the progression of chronic kidney disease (CKD) to end-stage kidney disease (1). Thus, dampening excessive and prolonged renal inflammation is an important therapeutic strategy for kidney disease progression and fibrosis. We previously demonstrated that homeodomain interacting protein kinase-2 (HIPK2), a signaling effector and transcriptional regulator with pleiotropic biological functions, is a key instigator of tubular cell injury, inflammation, and renal fibrosis in vivo (2, 3). HIPK2 is a multifunctional serine/threonine kinase, which acts as a transcriptional coactivator or corepressor in a cell- and context-dependent manner (4–6). In the context of kidney disease, we demonstrated that increased HIPK2 activity induces tubular cell injury and apoptosis through activation of the p53 signaling pathway and promotes renal fibrosis through enhancing TGF-β/Smad3, Notch, and Wnt/β-catenin signaling pathways (2, 3). HIPK2’s ability to control multiple signaling and transcriptional mediators is partly attributed to the multiple regions that participate in protein-protein interactions (7, 8). Given its wide-ranging roles in tissue homeostasis and injury with multiple protein interactions, pan-inhibition of HIPK2 may yield unwanted side effects, such as pro-oncogenic effects caused by p53 suppression in dampening renal fibrosis in CKD. Thus, a better understanding of the molecular mechanisms and HIPK2 regions involved in individual signaling pathways is needed to develop optimal HIPK2 inhibitors.

In addition to various protein-interacting regions, HIPK2 activity and stability are extensively regulated by various posttranslational modifications, one of which includes the caspase-mediated processing of HIPK2’s C-terminal region, referred to as the autoinhibitory domain (7, 8). In response to DNA damage, activation of p53 induces the expression of caspase-6, which cleaves HIPK2 at D923 and D984, releasing a short 275– or 214–amino acid fragment. While the removal of its C-terminal fragment is not required for the kinase activity of HIPK2 per se, HIPK2 cleavage leads to enhanced tumor-suppressive p53 response and apoptosis in cancer cells (9–11) and interferon-stimulated antiviral response in peritoneal macrophages (12). However, in muscle cells, HIPK2 cleavage results in reduced transcriptional repression of the myogenic differentiation program, as a result of reduced affinity of truncated HIPK2 to the transcriptional repressive protein complex (13). Similarly, the C-terminal portion of HIPK2 is required to recruit transcriptional corepressor CtBP and suppress β-catenin–mediated transcription necessary for regulation of mouse embryonic fibroblast proliferation (14) and associated with increased repression of YAP/TEAD-mediated gene transcription in cancer cell line, NSCLC (15). Interestingly, caspase-6–mediated processing of HIPK2 has been shown to occur in the nucleus, rather than in the cytoplasm (12). These results highlight the importance of specificity of interaction between HIPK2 and transcriptional coactivator or corepressor complexes and variegated biological response to HIPK2 cleavage in a cell-type and context-specific manner.

We and others also previously demonstrated that the increased HIPK2 activation leads to enhanced NF-κB–mediated inflammation in tubular cells in vitro and in mouse kidneys in vivo (2, 16). However, somewhat discordant HIPK2 roles in the regulation of NF-κB–mediated gene expression have been described in macrophages (i.e., enhancing and dampening) (17). Moreover, whether caspase-6–mediated HIPK2 processing has any role in kidney cell injury or renal fibrosis has not been previously explored. Our results now show that caspase-6–mediated release of C-terminal fragment enhanced HIPK2’s ability to activate NF-κB–mediated gene expression in vitro. Unexpectedly, we also found that the small C-terminal fragment of HIPK2 (HIPK2-CT) released by activated caspase-6 interacts with NF-κB signaling components, such that it paradoxically can attenuate p65 NF-κB phosphorylation and activity through cytoplasmic sequestration in renal tubular cells. Thus, we further explored whether the inhibitory effects of HIPK2-CT can be leveraged to suppress NF-κB–induced inflammation in vivo. Indeed, the overexpression of HIPK2-CT in kidney tubular cells was sufficient to attenuate the expression of inflammatory cytokines and macrophage infiltration in the kidneys of mice with unilateral ureteral obstruction (UUO) and LPS-induced acute kidney injury (AKI), demonstrating that HIPK2-CT can be further exploited as a therapeutic approach to restrain renal inflammation in vivo.

## Results

### HIPK2-CT is released by active caspase-6 in kidney cells.

**Figure 1:**
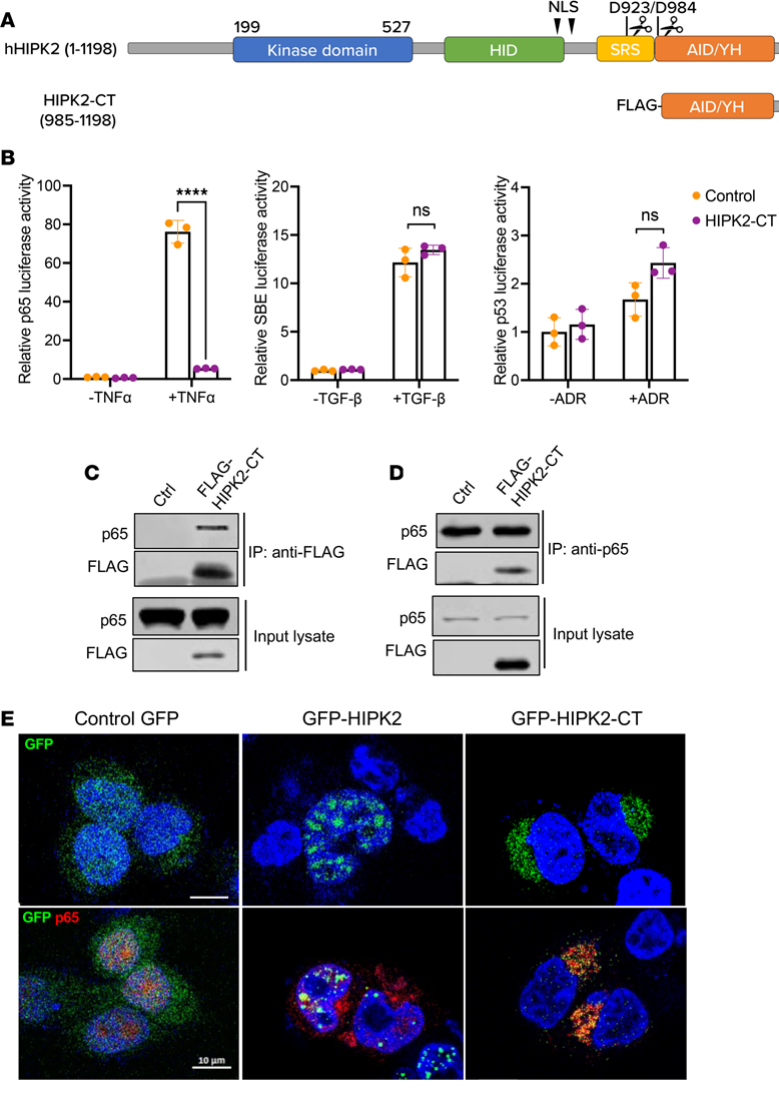
HIPK2-CT abrogates p65 NF-κB signaling in HEK293T cells. (**A**) The schematics shows WT full-length human HIPK2 structure and FLAG-tagged HIPK-CT deletion mutant. Nuclear localization signals (NLS) and caspase-6 cleavage sites on D923 and D984 are indicated. Amino acids 1–1,198 and 985–1,198 are shown. (**B**) The effects of HIPK2-CT were examined on transcriptional activities of p65 NF-κB, Smad3, or p53 by luciferase reporters in HEK293T cells. Cells transiently transfected with luciferase vectors were treated with 10 ng/mL TNF-α, 10 ng/mL TGF-β1, or 0.5 μg/mL adriamycin for 16 hours. Vehicle-treated cells served as negative controls. Values represent mean ± SD from 3 independent experiments. ****P < 0.0001 between indicated groups by 2-way ANOVA with Tukey’s correction. (**C**) HEK293T cells were transiently transfected with control (Ctrl) or FLAG-tagged HIPK2-CT plasmid, and lysates were immunoprecipitated with anti-FLAG antibody and immunoblotted using anti-p65 and anti-FLAG antibodies. (**D**) The same lysates were used for immunoprecipitation with anti-p65 antibody and immunoblotted with anti-p65 and anti-FLAG antibodies. Input lysates were also immunoblotted to show the expression of FLAG-tagged proteins. (**E**) HEK293T cells cotransfected with mCherry-p65 and GFP-HIPK2 or GFP-HIPK2-CT were imaged with confocal microscopy. The top row shows representative examples of GFP proteins, and the bottom row shows both mCherry-p65 and GFP proteins. DNA was counterstained with DAPI. Scale bar: 10 μm.

As shown in the schematics in Figure 1A, HIPK2 protein architecture consists of a kinase domain near the N-terminus; homeoprotein interacting domain (HID), which is conserved among HIPK proteins (HIPK1–HIPK3); speckled-retention sequence (SRS), which is responsible for HIPK2’s subcellular localization in the nuclear bodies; C-terminal region encompassing an autoinhibitory domain, which leads to hyperactive p53 activation when released by caspase-mediated cleavage on D923 and D984; and YH domain, which has been shown to interact with multiple transcriptional regulators (7, 8). We first confirmed that HIPK2 undergoes caspase-6–mediated processing in kidney cells. HEK293T cells were transiently transfected with plasmids expressing V5-tagged full-length HIPK2 and caspase-6 or a cleaved form of caspase-6 (active caspase-6). As expected, overexpression of WT or cleaved caspase-6 released smaller V5-tagged HIPK2 fragments (Supplemental Figure 1, A and B; supplemental material available online with this article; https://doi.org/10.1172/jci.insight.175153DS1).

To ascertain the HIPK2 regions involved in RelA/p65 NF-κB signaling, we made a series of HIPK2 mutants (Supplemental Figure 1B) and compared the effects of each mutant to HIPK2 full-length expression (HIPK2-WT) on p65 NF-κB luciferase activity in HEK293T cells. Following TNF-α stimulation, while the kinase-dead HIPK2 mutant (HIPK2-K228R) or the mutant bearing the deletion of the Smad-interacting domain (HIPK2-ΔSID) did not alter the p65-luciferase activity, the deletion of the C-terminal region released by caspase-6 cleavage substantially enhanced p65 activity (Supplemental Figure 1C). These results suggested that the removal of the purported autoinhibitory region of HIPK2 also enhanced the p65 activity.

Moreover, the expression of a small C-terminal fragment that correlates with a fragment released by caspase-6 cleavage (corresponding to amino acid residues 984–1,198; referred to as HIPK2-CT hereafter) nearly abrogated the p65 activity in TNF-α–treated cells (Figure 1, A and B). As HIPK2-CT lacks the HIPK2 kinase domain, it did not affect TGF-β–induced Smad3 or adriamycin-induced p53 transcriptional activity (Figure 1B), as a full-length HIPK2 protein would (2). We confirmed that HIPK2-CT indeed interacts with p65 with a coimmunoprecipitation experiment using HEK293T cells with FLAG-tagged HIPK2-CT overexpression. Anti-FLAG beads used to capture FLAG-HIPK2-CT also pulled down p65 in HEK293T lysates (Figure 1C), and conversely, anti-p65 antibody pulled down FLAG-tagged HIPK2-CT (Figure 1D), indicating that HIPK2-CT interacts with p65 or p65-containing protein complex.

In cells, HIPK2 is found mainly within the subnuclear speckles via its nuclear localization signal sequences and speckle retention signal (Figure 1A). As HIPK2-CT lacks these sequences and the above data indicated the interaction between HIPK2-CT and p65, we next assessed the subcellular localization of HIPK2-CT and its effects on p65. HEK293T cells were transfected with mCherry-p65 and GFP-HIPK2 (full length), GFP-HIPK2-CT, or control GFP expression vectors, and fluorescent proteins were visualized with confocal microscopy. As expected, the control GFP protein showed a diffuse localization, full-length GFP-HIPK2 localized to discrete speckles in the nucleus, and HIPK2-CT aggregated predominantly in the cytoplasm (Figure 1E). Following TNF-α treatment, a large portion of mCherry-p65 colocalized with HIPK2 in the nuclear speckles (Figure 1E), although its expression was not limited to the nuclear bodies. Notably, in cells expressing GFP-HIPK2-CT, no nuclear localization of mCherry-p65 was observed after TNF-α treatment. Together with the above results, these results suggested HIPK2-CT interacts with p65 and/or p65-containing protein complex and that, when overexpressed, it can act as a decoy to sequester p65 and inhibit its nuclear translocation and downstream transcriptional activity.

### HIPK2-CT reduces p65 phosphorylation and stabilizes the IκB-p65 complex.

Under basal conditions, NF-κB signaling is kept inactive by NF-κB dimers complexed with IκB inhibitory proteins that block their nuclear import (18). Upon cytokine stimulation, IκB is rapidly phosphorylated and degraded to release p65 for nuclear import, and p65 phosphorylation, such as on Ser536 in the transactivation domain, promotes its activation and nuclear import. Therefore, we next assessed the effects of HIPK2-CT on IκB phosphorylation and degradation and on p65 phosphorylation on Ser536.

**Figure 2:**
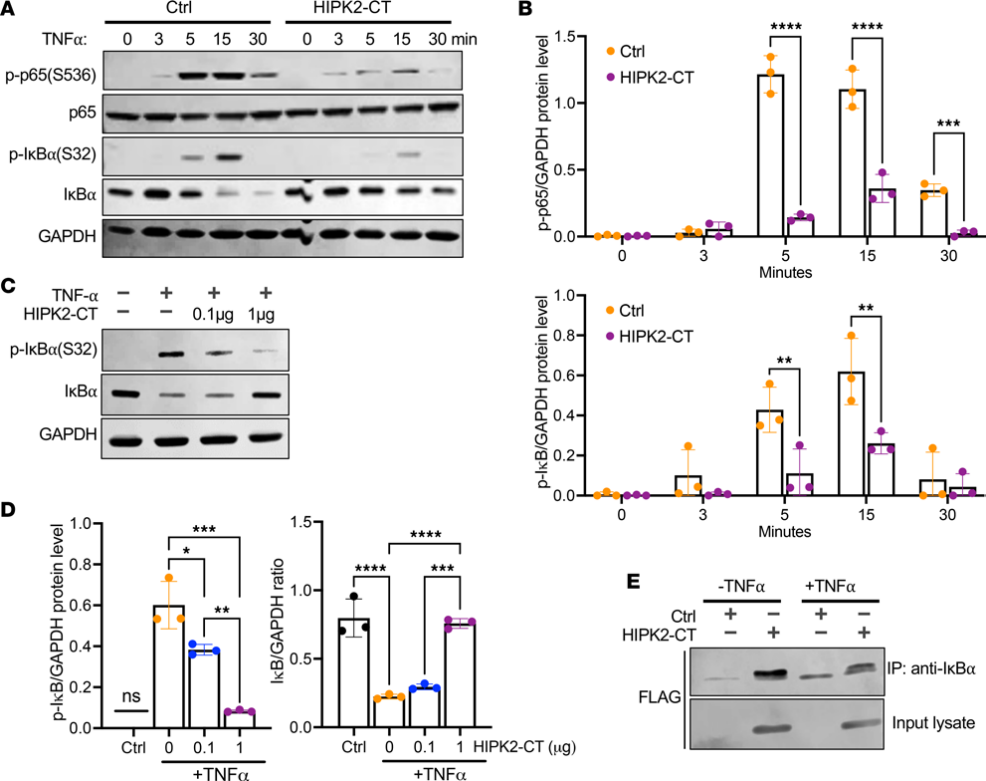
HIPK2-CT reduces TNF-induced p65 phosphorylation and IκB degradation. (**A**) HEK293T cells transfected with control (Ctrl) or FLAG-tagged HIPK2-CT plasmids (1 μg) were stimulated with 10 ng/mL TNF-α for indicated times (0–30 minutes). Cell lysates were probed for phospho-p65 (S536), total p65, phospho-IκB (S32), and total IκB. GAPDH was used as an internal control. (**B**) Densitometric analysis of p-p65 (S536) and phospho-IκB (S32) levels (normalized to GAPDH). Values represent mean ± SD from 3 independent experiments. **P < 0.01, ***P < 0.001, ****P < 0.0001 between indicated groups by 2-way ANOVA with Bonferroni’s correction. (**C**) HEK293T cells transfected with 0, 0.1, or 1 μg HIPK2-CT plasmid were treated with 10 ng/mL TNF-α for 15 minutes. Cell lysates were probed for phospho-IκB (S32) and total IκB. (**D**) Densitometric analysis of normalized phospho-IκB (S32) and total IκB levels. Values represent mean ± SD from 3 independent experiments. *P < 0.05, **P < 0.01, ***P < 0.001, ****P < 0.0001 between indicated groups by 1-way ANOVA with Tukey’s correction. (**E**) Lysates of HEK293T cells transfected with FLAG-tagged HIPK2-CT were immunoprecipitated with anti-IκBα antibody and immunoblotted with anti-FLAG antibody. Input lysates were also immunoblotted to show the expression of FLAG-tagged proteins.

Following TNF-α stimulation, p65 and IκBα were rapidly phosphorylated, and this peaked between 5 and 15 minutes (Figure 2, A and B). In HIPK2-CT–expressing cells, although a similar kinetics of phosphorylation of p65 was observed, there was a marked reduction in overall p65 phosphorylation that was independent of p65 protein degradation (Figure 2, A and B, and Supplemental Figure 3). HIPK2-CT also markedly reduced TNF-α–mediated IκBα phosphorylation (Figure 2, A and B) and prevented its degradation (Figure 2, A and B, and Supplemental Figure 3). This effect on reduced IκBα degradation was further confirmed by varying the levels of HIPK2-CT. As shown in Figure 2, C and D, increasing HIPK2-CT plasmid concentration in transfected cells resulted in a reduction in IκBα phosphorylation and increased protein stability. We further confirmed the interaction between HIPK2-CT and IκBα using coimmunoprecipitation methods, which showed the pull-down of IκBα by immunoprecipitation of FLAG-tagged HIPK2-CT (Figure 2E). Together, these results indicate that HIPK2-CT interacts with the IκBα-p65 complex, thereby preventing the IκBα degradation and p65 nuclear import.

### Tubular cell–specific overexpression of HIPK2-CT attenuates renal inflammation in obstructive AKI.

Leveraging the inhibitory capacity of HIPK2-CT to reduce p65 NF-κB–mediated gene expression, we explored whether the expression of HIPK2-CT could dampen renal inflammation in vivo. To do so, we generated a double-transgenic mouse model that expresses human HIPK2-CT sequence under the tetracycline-responsive element (TRE) and *Pax8* promoter-driven reverse tetracycline-dependent transactivator (Pax8-rtTA), which results in the inducible transgene expression in all proximal and distal tubules and the collecting duct system (19) (Supplemental Figure 2A). As expected, the administration of tetracycline analog, doxycycline (dox), led to the induction of HIPK2-CT expression in all renal tubular cells (Supplemental Figure 2B). To assess the effects of HIPK2-CT in sterile inflammation associated with obstructive AKI, UUO was performed in double-transgenic mice with dox induction (referred to as Pax8-HIPK2-CT mice) or control littermates with 1 or no transgene expression with dox induction (referred to as WT mice). Contralateral sham-operated kidneys served as negative controls. Mouse kidneys were examined 3 days after UUO, because the obstructed kidneys are typically characterized by marked tubulointerstitial lesion and inflammation at this time point, and progressively worsening kidney damage and fibrosis are observed by 7–14 days after UUO (20).

**Figure 3:**
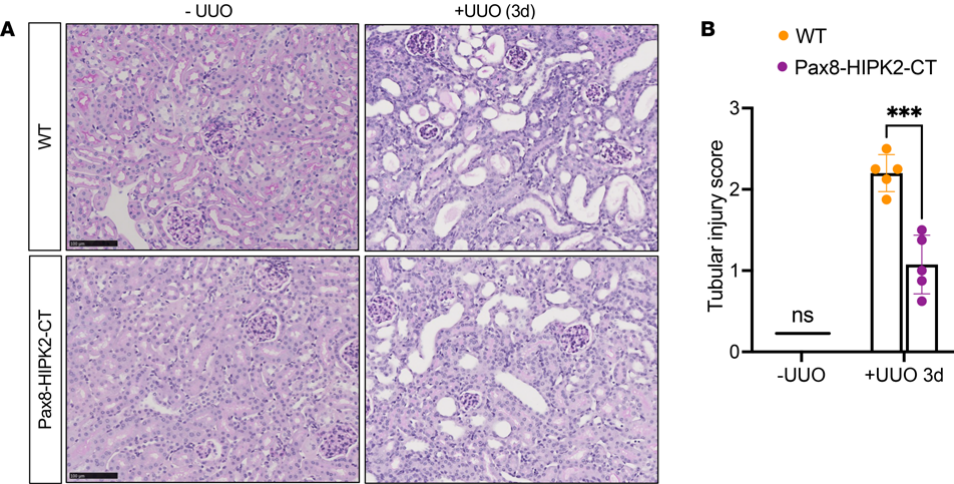
Tubular cell–specific overexpression of HIPK2-CT attenuates tubular injury in obstructive AKI. Control WT and Pax8-HIPK2-CT mice were subjected to unilateral ureteral obstruction (UUO), and kidneys were analyzed after 3 days after surgery. Sham-operated contralateral kidneys (-UUO) served as negative controls. (**A**) Representative images of periodic acid–Schiff–stained kidneys. Scale bar: 100 μm. (**B**) Quantification of tubular injury score (scale of 0–4) for individual mouse kidneys (*n* = 5 mice per group, 20 fields per kidney analyzed). ***P < 0.001 between indicated groups by 1-tailed *t* test with Welch’s correction.

**Figure 4:**
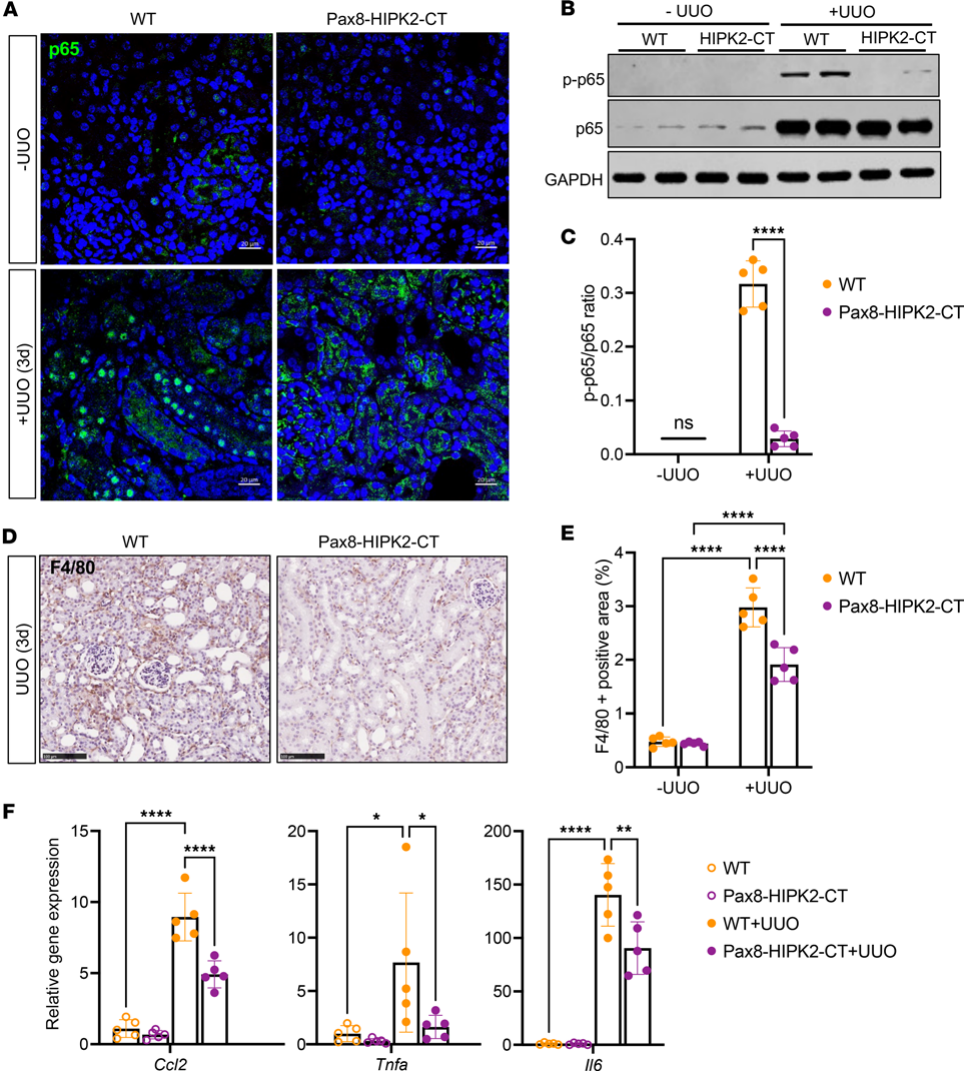
Tubular cell–specific overexpression of HIPK2-CT attenuates renal inflammation in obstructive AKI. (**A**) Representative immunofluorescence image of p65 expression in kidneys of mice 3 days after UUO. Scale bar: 20 μm. (**B**) Western blot analysis of phospho-p65 (S536) and total p65 in kidney cortices of mice in each group (*n* = 2 per group are shown). (**C**) Densitometric analysis of p-p65 to total p65 is shown for each mouse (*n* = 5). ****P < 0.0001 between indicated groups by 1-tailed *t* test with Welch’s correction. (**D**) Representative immunohistochemical images of F4/80 expression in mouse kidneys. Scale bar: 100 μm. (**E**) Quantification of F4/80^+^ area per mouse kidney (*n* = 5 mice per group, 20 fields per kidney analyzed). ****P < 0.0001 between indicated groups by 2-way ANOVA with Tukey’s correction. (**F**) Quantitative PCR analysis of *Ccl2*, *Tnfa,* and *Il6* transcripts in kidney cortices (*n* = 5 mice per group). *P < 0.05, **P < 0.01, ****P < 0.0001 between indicated groups by 1-way ANOVA with Dunnett’s correction.

While the induction of HIPK2-CT expression in tubular cells had no effect on sham-operated kidneys, it led to a significant reduction in tubular injury in obstructed kidneys (Figure 3). This was associated with a reduction of nuclear localization of p65 (Figure 4A) and p65 (Ser536) phosphorylation (Figure 4, B and C), although the total level of p65 in obstructed kidneys of all mice was comparably elevated in comparison with that of sham kidneys. Concomitantly, there was a marked reduction in the infiltration of F4/80^+^ macrophages in the obstructed kidneys of Pax8-HIPK2-CT mice (Figure 4, D and E), and the expression of inflammatory cytokines, C-C motif chemokine ligand 2 (*Ccl2*), *Tnfa*, and *Il6* (Figure 4F). These results are consistent with the above in vitro observation that HIPK2-CT hinders the p65 NF-κB pathway to reduce inflammation in tubular cells.

### Tubular cell–specific overexpression of HIPK2-CT attenuates renal fibrosis in obstructed kidneys.

**Figure 5:**
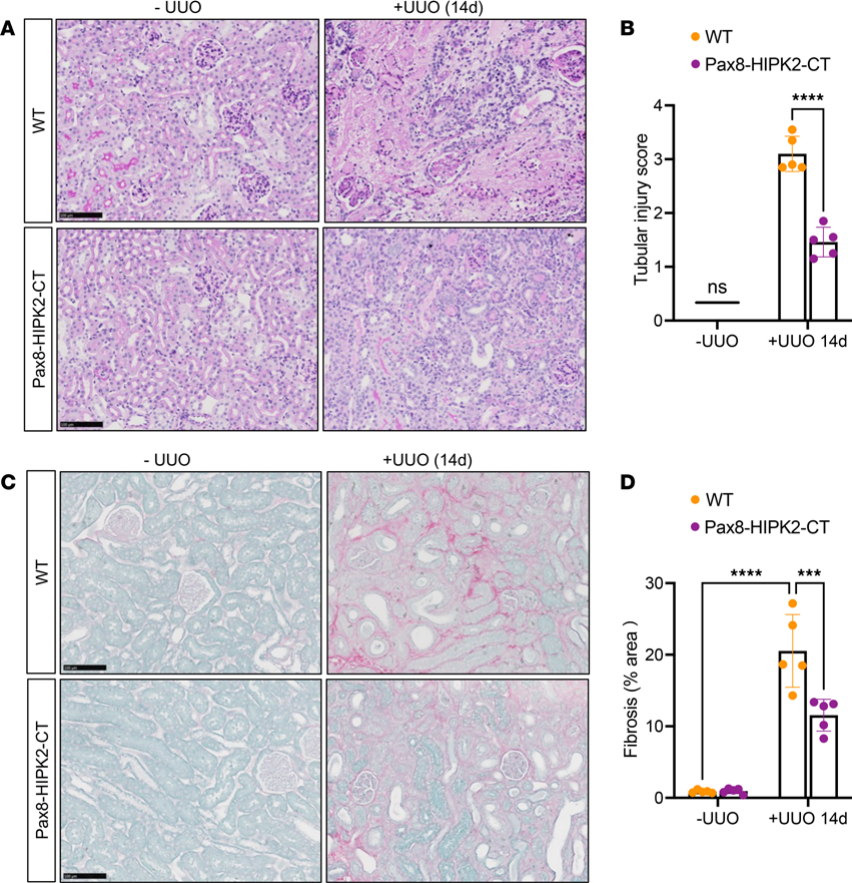
Tubular cell–specific overexpression of HIPK2-CT attenuates UUO-induced renal fibrosis. Control WT and Pax8-HIPK2-CT mice were subjected to unilateral ureteral obstruction (UUO), and kidneys were analyzed after 14 days after surgery. Sham-operated contralateral kidneys (-UUO) served as negative controls. (**A**) Representative images of periodic acid–Schiff–stained kidneys. Scale bar: 100 μm. (**B**) Quantification of tubular injury score (scale of 0–4) for individual mouse kidneys. ****P < 0.0001 between indicated groups by 1-tailed *t* test with Welch’s correction. (**C**) Representative images of Picrosirius Red– and Fast Green–stained mouse kidneys. Scale bar: 100 μm. (**D**) Quantification of percentage fibrosis area relative to total kidney area (*n* = 5 mice per group, 10 fields analyzed per mouse). ***P < 0.001, ****P < 0.0001 between indicated groups by 2-way ANOVA with Tukey’s correction.

We next assessed whether the reduced renal inflammation in the obstructed kidneys can attenuate the ensuing renal fibrosis. Using a similar experimental approach as above, we assessed the tubular injury and renal fibrosis after 14 days after surgery. Compared with the sham-operated kidneys, the 14-day UUO kidneys of both WT and Pax8-HIPK2-CT mice displayed marked tubular injuries; however, the extent of tubular injury was substantially reduced in Pax8-HIPK2-CT UUO kidneys in comparison with that in WT UUO kidneys (Figure 5, A and B). Similarly, fibrosis development was significantly dampened in Pax8-HIPK2-CT UUO kidneys in comparison with that in WT UUO kidneys (Figure 5, C and D). These results indicate that reduced renal inflammation in Pax8-HIPK2-CT kidneys can slow fibrosis progression and associated tubular injury.

### Tubular cell–specific overexpression HIPK2-CT attenuates LPS-induced AKI.

**Figure 6:**
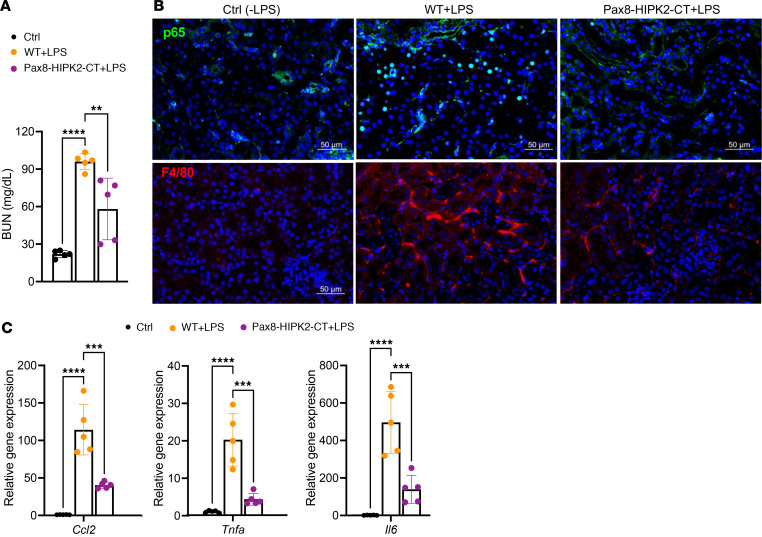
Tubular cell overexpression of HIPK2-CT attenuates LPS-induced tubular injury. (**A**) Blood urea nitrogen (BUN) levels of mice after 24 hours of LPS or vehicle (Ctrl) injection (*n* = 5 mice per group). **P < 0.01, ****P < 0.0001 between indicated groups by 1-way ANOVA with Dunnett’s correction. (**B**) Representative immunofluorescence images of p65 (green) and F4/80 (red). DNA was counterstained with DAPI. (**C**) Quantitative PCR analysis of *Ccl2*, *Tnfa,* and *Il6* transcripts in kidney cortices (*n* = 5 mice per group). ***P < 0.001, ****P < 0.0001 between indicated groups by 1-way ANOVA with Dunnett’s correction.

To further validate the protective role of HIPK2-CT in renal injury and inflammation, we next examined its efficacy to curb kidney injury in the LPS-induced AKI model. A single dose of LPS was intraperitoneally administered in WT or Pax8-HIPK2-CT mice after 5 weeks of dox induction, and kidneys were examined 24 hours after injection. Mice given saline injection were used as negative controls. As expected, LPS injection resulted in elevated blood urea nitrogen (BUN) levels in all mice; however, Pax8-HIPK2-CT mice showed significantly lower BUN levels than WT mice after LPS injection (Figure 6A), indicative of attenuation of LPS-induced tubular injury by HIPK2-CT. Similar to the above acute obstructive injury, we also observed reduced F4/80^+^ macrophage infiltration in the kidneys of Pax8-HIPK2-CT mice in comparison with those of WT mice (Figure 6B) and reduced expression of inflammatory cytokines (Figure 6C). Taken together, our data indicate that HIPK2-CT could hinder p65 nuclear translocation and gene expression to dampen renal inflammation following AKI (Supplemental Figure 4).

## Discussion

Using the unbiased systems biology approach, we previously identified HIPK2 as a pivotal signaling modulator of kidney disease and fibrosis progression (2). HIPK2 acts as a signaling effector and transcriptional coactivator/corepressor to dictate various biological responses. In CKD, its expression is increased in kidney epithelial cells, and knocking out HIPK2 in mice (global or tubular cell specific) effectively reduces renal fibrosis and improves kidney function (2, 3). However, given its pleiotropic role, including a well-established tumor suppressive function, a complete blockade of HIPK2 as a long-term therapeutic approach in CKD may yield unwanted side effects. In this manner, we recently developed a small-molecule HIPK2 inhibitor that allosterically interferes with TGF-β–induced Smad3 activity to reduce renal fibrosis in vivo, without affecting the kinase function of HIPK2 and altering its influence on p53 (21).

One of the key drivers of CKD pathogenesis is the unregulated excessive inflammatory response, involving both immune and nonimmune cells, such as tubular cells that actively contribute to the process by inflammatory cytokine/chemokine production (22, 23). While inflammatory mediators invariably trigger the activation of NF-κB signaling, its transduction is important for both the initiation and resolution phases of inflammation, and thus, too much and too little signaling are both detrimental (24, 25). Moreover, NF-κB signaling has crucial roles not only in the regulation of inflammatory response, but also in cell proliferation, differentiation, and survival (25–27). Thus, a better understanding of the mechanism underlying the pathological activation of NF-κB in kidney injury is crucial for designing more specific and effective therapeutic agents for CKD and renal fibrosis.

In the current study, we have identified the C-terminal region that is involved in the repression of p65 NF-κB gene expression, and when overexpressed, it can act to sequester p65 in the cytoplasm to dampen its activity. We confirmed that the overexpression of HIPK2-CT in renal tubular cells can attenuate the excessive renal inflammation and tubular injury triggered by UUO and LPS and consequently reduce tubulointerstitial fibrosis in obstructed kidneys. While precise amino acid sequences required for this interaction within the C-terminal fragment are not yet determined, these data suggest that HIPK2-CT analogs could be potentially developed as inhibitors of NF-κB and drugs to curb renal inflammation without a complete blockade. Interestingly, HIPK2 has been shown to restrain NF-κB activation in macrophages via interaction with histone deacetylase 3 (17). Given that HIPK2-CT expression reduced renal fibrosis in obstructed kidneys after 14 days after UUO, it may be plausible to target the overactive p65 activation in the setting of chronic renal inflammation by leveraging the actions of HIPK2-CT.

We further confirmed that caspase-6 mediates the cleavage of HIPK2 to release the C-terminal fragment in kidney cells. Caspase-6 expression is typically induced in cells under genotoxic stress by the activated p53 to induce apoptosis. A recent report of HIPK2 cleavage in macrophages suggests that caspase-6–mediated cleavage of HIPK2 occurs following its translocation to the nucleus, which endows enhanced transcriptional activity for truncated HIPK2 (12). Although it is not yet known whether HIPK2 is cleaved after its nuclear import in renal tubular cells, our data indicate that the overexpression of the C-terminal fragment that lacks the nuclear localization signal sequence localizes to the cytoplasm, prohibiting the nuclear translocation of p65 in tubular cells in vitro and in vivo. We have confirmed the interaction of HIPK2-CT with p65 and IκBα by coimmunoprecipitation; further biochemical studies are necessary to assess whether these interactions are direct or indirect.

In summary, our findings suggest that HIPK2-CT, generated by active-caspase-6, can act as a negative regulator of renal inflammation by inactivation of the NF-κB pathway in renal tubular cells and that HIPK2-CT peptide analogs could be potentially developed to modulate NF-κB and antiinflammatory response in CKD.

## Methods

### Sex as a biological variable.

Our study examined male mice, because male animals exhibited less variability in phenotype.

### Expression constructs.

The expression constructs for V5-tagged hHIPK2 were previously described (3, 21). Flag-HIPK2-CT, V5-HIPK2-CT, GFP-HIPK2, GFP-HIPK2-CT, mCherry-p65, caspase-6, and cleaved caspase-6 (amino acids 193–294) were generated using a PCR-based standard procedure, and detailed information and primers used to generate cDNA for each expression constructs are described in the Supplemental Methods.

### Cell culture and transfection.

HEK293T cells were obtained from ATCC and cultured according to the manufacturer’s specifications in DMEM medium (Gibco) containing 10% fetal bovine serum and 1% penicillin-streptomycin (Gibco). For transient transfection, HEK293T cells were transfected using PolyJet reagent (SignaGen, SL100688).

### Luciferase reporter gene assays.

HEK293T cells were cotransfected with pcDNA4B or HIPK2-CT, as well as Renilla expression vector to normalize transfection efficiency, and p65 luciferase reporter construct or Smad3-binding element luciferase reporter construct or p53 luciferase reporter construct (p65-luc construct was a gift from the laboratory of Huabao Xiong [Department of Medicine, Precision Immunology Institute, Icahn School of Medicine at Mount Sinai], ref. 28, and SBE-luc and p53-luc were obtained from Addgene). Twenty-four to 36 hours after transfection, cells were stimulated with 10 ng/mL TNF-α (p65), 10 ng/μL TGF-β1 (Smad3), or 0.5 μg/mL adriamycin (p53) for 16 hours. The luciferase assays were performed with the Dual-Luciferase Reporter Assay Kit (Promega, E1910).

### Immunocytochemistry and confocal imaging.

HEK293T cells cotransfected with GFP-HIPK2-WT or GFP-HIPK2-CT and mCherry-p65 plasmids were treated with or without 10 ng/mL TNF-α for 30 minutes. Cells were then fixed with freshly made 4% formaldehyde (pH 7.5) for 15 minutes at room temperature. After a brief wash, DNA was counterstained with DAPI, and slides were mounted with the anti-fade medium (Vectashield, H1200). Images were acquired using Zeiss LSM880 Airyscan confocal microscope (Carl Zeiss).

### Western blot analysis.

Protein lysate preparation and Western blot analysis were performed according to the standard protocol using 30–60 μg total protein. For densitometric analysis, the density for each target protein was normalized to GAPDH or β-actin. The following antibodies were used in this study: anti-V5 (GenScript, A01724), anti-FLAG (MilliporeSigma, F3165), p-p65 (Cell Signal Technology, 3033), p65 (Cell Signal Technology, 8242), p-IκB (Cell Signal Technology, 2859), IκB (Abcam, ab32518), β-actin (Millipore Sigma, A4700), and GAPDH (Cell Signal Technology, A2118).

### Immunoprecipitation.

Cells were washed in ice-cold PBS and lysed in lysis buffer (Thermo Fisher Scientific, 87788) supplemented with a protease inhibitor cocktail (MilliporeSigma, P8340). After incubating on ice for 15 minutes with periodic mixing, cell lysates were centrifuged, collected, and incubated with the following antibodies/reagents for immunoprecipitation: anti-p65 antibody (1:50; Santa Cruz, sc8008), anti-IκB antibody (1:50; Santa Cruz, sc1643), protein G–agarose, or anti-FLAG mAb magnetic beads (MilliporeSigma, M8823).

### Mouse model.

Human HIPK2-CT cDNA corresponding to NM_022740.5 was subcloned with a C-terminal HA-tag into the pTRE-Tight vector (Clontech, 631059) expressing the TRE. The sequence of the HIPK2-CT insert was confirmed by restriction endonuclease and DNA sequencing. The linearized DNA fragment of pTRE-Tight-hHIPK2-CT-HA was used for microinjection to generate the TRE-HIPK2-CT–transgenic mice in the FVB/NJ background. To generate tubular epithelial cell–specific overexpression of HIPK2-CT mice, TRE-HIPK2-CT–transgenic mice were crossed with Pax8-rtTA–transgenic mice, which express the reverse tetracycline-controlled trans-activator (rtTA) protein under the control of murine *Pax8* promotor, which directs the expression in all renal tubular cells (19). Tubular cell–specific HIPK2-CT expression was induced by the administration of dox-supplemented chow (625 g/kg chow, Envigo).

The UUO model was created according to a previous protocol (20). Briefly, the left ureter of each mouse was exposed through a midline abdominal incision and ligated using 4.0 silk. Contralateral sham-operated kidneys (exposure of ureters, without ligation) were used as controls. All surgeries were performed under general anesthesia with isoflurane. To identify renal inflammation in acute injury and fibrosis in chronic kidney injury, mice were sacrificed 3 days and 14 days after UUO surgery. Kidneys were perfused in situ with PBS, and tissue samples from both kidneys were collected for histology and Western blot analysis.

For LPS-induced AKI, mice were administrated as described previously (29, 30). Age- and sex-matched WT and Pax8-HIPK2-CT mice received a single low dose of LPS (5 mg/kg) injection intraperitoneally. Twenty-four hours after injection, serum and samples were harvested and processed for BUN and immunostaining.

### Mouse kidney histology.

Kidney tissues were fixed in 10% formalin, embedded in paraffin, and sectioned to 4 μm thickness. Periodic acid–Schiff–stained and Sirius Red/Fast Green-stained kidney sections were used for kidney histology. Histological scoring was performed by the renal pathologists, with experimental groups and mouse genotypes masked. To evaluate tubular injury score, 10 random tissue sections (×20 magnification) per mouse were assessed for PAS staining and scored semiquantitatively as follows: 1, none; 1, <25%; 2, 25%–50%; 3, 50%–75%; 4, >75%. Results are presented as average for each mouse. To evaluate interstitial fibrosis, 10 tissue sections per mouse were obtained randomly (×20 magnification) and assessed after Sirius Red/Fast green staining (Chondrex, 9046). Tissue fibrosis, as defined by red staining, was scored, and the average values of the fibrosis scores are reported.

### Immunohistochemical and immunofluorescence staining.

Immunofluorescence staining was conducted on frozen sections using standard procedures using the anti-p65 antibody (Cell Signal Technology, 8242) or anti-F4/80 antibody (Abcam, ab6640), nuclei were counterstained with DAPI, and slides were mounted with anti-fade medium (Vectashield, H1200).

For immunohistochemical staining, deparaffinized kidney sections were incubated with anti-F4/80 antibody (1:100) and processed using the Vectastain Elite ABC kit (Vector Laboratories, PK-6100). Images were acquired using an AxioVision II microscope with a digital camera (Carl Zeiss).

### Quantitative real-time PCR assay.

The total RNA from cells or kidney cortex was extracted using TRIzol (Life Technologies). The cDNA was then synthesized using a PrimeScript RT reagent kit (Takara, RR037A). qPCR was performed with the TB Green Premix Ex Taq kit (Takara, RR420A) using a 7500 real-time PCR System (Applied Biosystems). All the primer sequences are listed in Supplemental Methods.

### Statistics.

Data are presented as the mean ± SD. Comparison between 2 groups was performed with a 1-tailed, unpaired *t* test. Three or more groups were analyzed with 1-way or 2-way ANOVA, as appropriate. All statistical analyses were performed using GraphPad Prism software (version 9). *P* values of less than 0.05 were considered statistically significant.

### Study approval.

All mouse protocols were approved by the Institutional Animal Care and Use Committee at Icahn School of Medicine at Mount Sinai (no. LA09-00377).

### Data availability.

All the data are available from the corresponding author upon request. Values for all data points in graphs are reported in the Supporting Data Values file.

## Author contributions

YF, BCL, KL, and JCH conceived and designed the experiments. YF, ZL, and HW conducted and acquired the data. YF, KL, and JCH analyzed the data. YF, KL, and JCH drafted and revised the manuscript. All authors approved the final version of the manuscript.

## Supplementary Material

Supplemental data

Unedited blot and gel images

Supporting data values

## Version 1 03/21/2024

In-Press Preview

## Version 2 04/22/2024

Electronic publication
