# Supplementary material for: HIPK2 C-terminal domain inhibits NF-κB signaling and renal inflammation in kidney injury
Source: JCI Insight. 2024 Mar 21;9(8):e175153. doi: 10.1172/jci.insight.175153 (PMC11141872; doi:10.1172/jci.insight.175153)
Supplement: Supplemental data [file jciinsight-9-175153-s185.pdf]

Supp. Figure 1

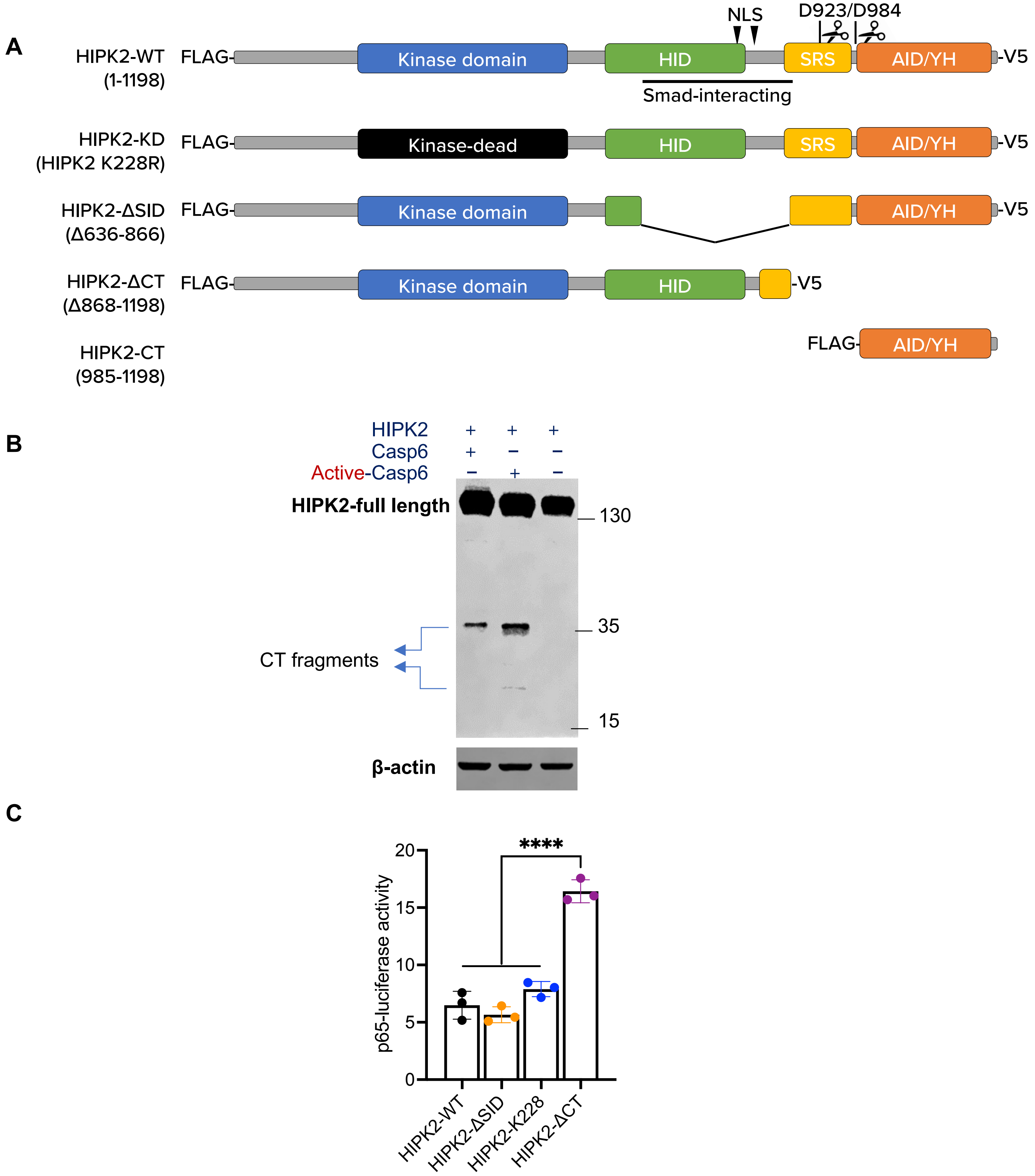

Supp. Figure 1: HIPK2 architecture and domain mutants. (A) Human HIPK2 cDNA was subcloned into expression vectors as full-length wildtype (WT), kinase-dead (KD) mutant, deletion mutant lacking the putative Smad-interacting region (ΔSID), deletion mutant lacking C-terminal region (ΔCT), or C-terminal fragment (CT). HID, homeobox-interacting domain. SRS, speckle-retention signal. AID, auto-inhibitory domain. (B) HEK293T cells were co-transfected with V5-tagged HIPK2-WT and Caspase 6 (full-length or cleaved active form), and lysates were probed with anti-V5 antibody. (C) HEK293T cells co-transfected with HIPK2 expression vectors and luciferase vectors (p65-responsive firefly luciferase and renilla luciferase) were treated with TNF-α for 16 hours. p65-luciferase activity normalized to renilla luciferase activity is shown. Representative results from 3 independent experiments. ****P<0.0001 between indicated groups by 1-way ANOVA with Tukey's modification.

Supp. Figure 2

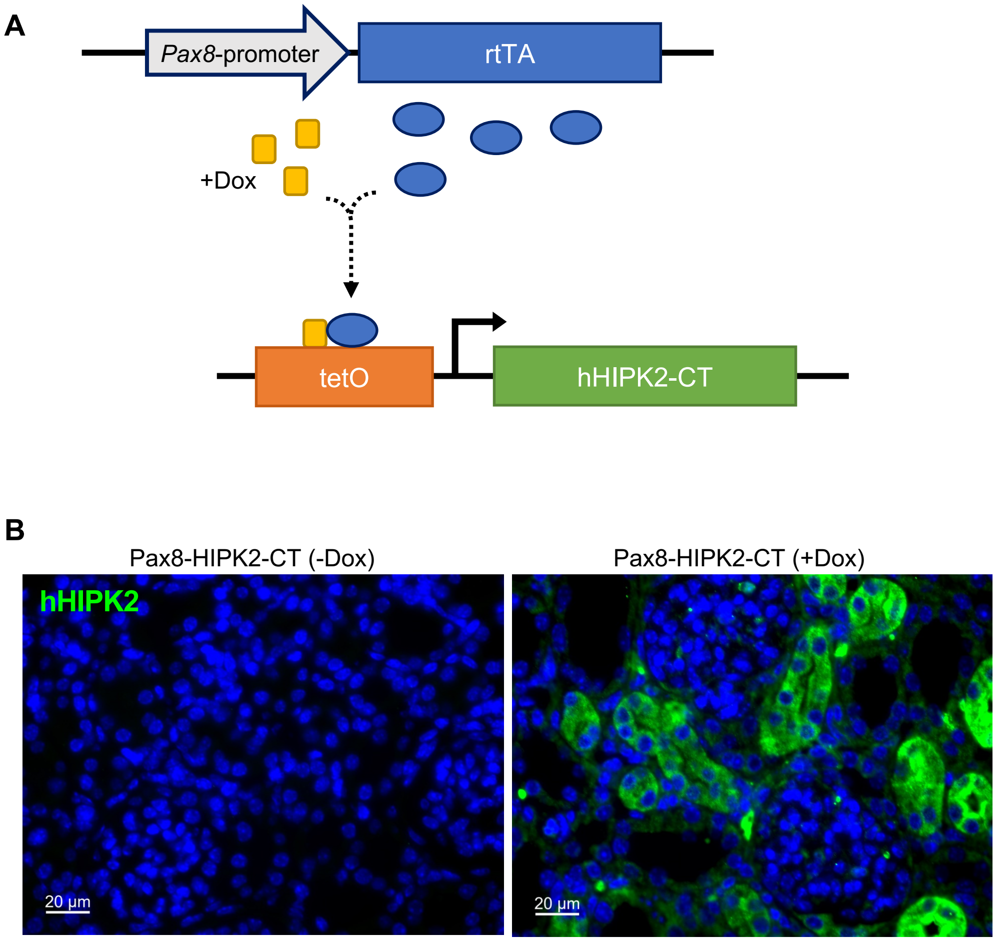

Supp. Figure 2: Induction of HIPK2-CT expression in renal tubules of transgenic mice. (A) Schematics of *Pax8*+ tubular cell-specific HIPK2-CT transgene expression. *Pax8* promoter directs the expression of reverse tetracycline transactivator (rtTA), which binds and activates the teracycline-responsive element (tetO) in presence of doxycyline (Dox) to drive the expression of HIPK2-CT. (B) Representative immunofluorescence imaging of HA-tagged HIPK2-CT using anti-HA antibody (green). DNA is counterstained with DAPI (n=5 mice per group, at least 10 fields examined per kidney).

Supp. Figure 3

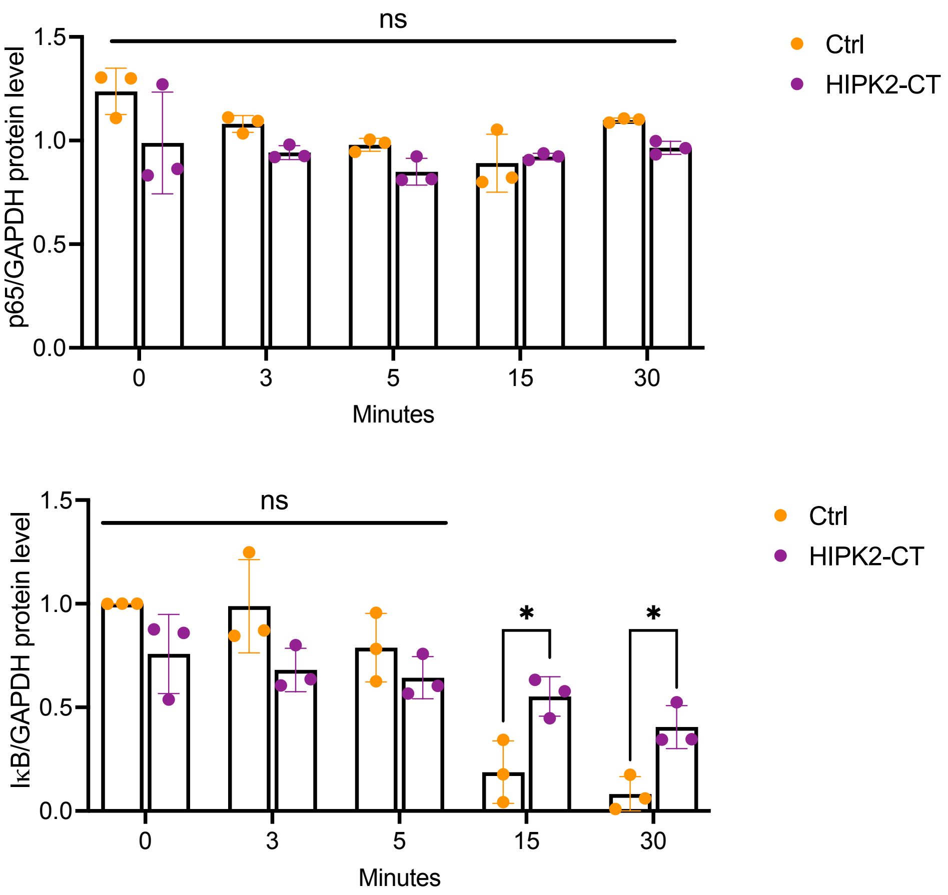

Supp. Figure 3: HIPK2-CT does not reduce NF-κB p65 and IκB-α protein levels. Densitometric analysis of normalized total p65 and IκBα levels from Figure 2A. Values represent mean±SD from 3 independent experiments. **P<0.01 and ****P<0.0001 between indicated groups by two-way ANOVA with Bonferroni's correction.

Supp. Figure 4

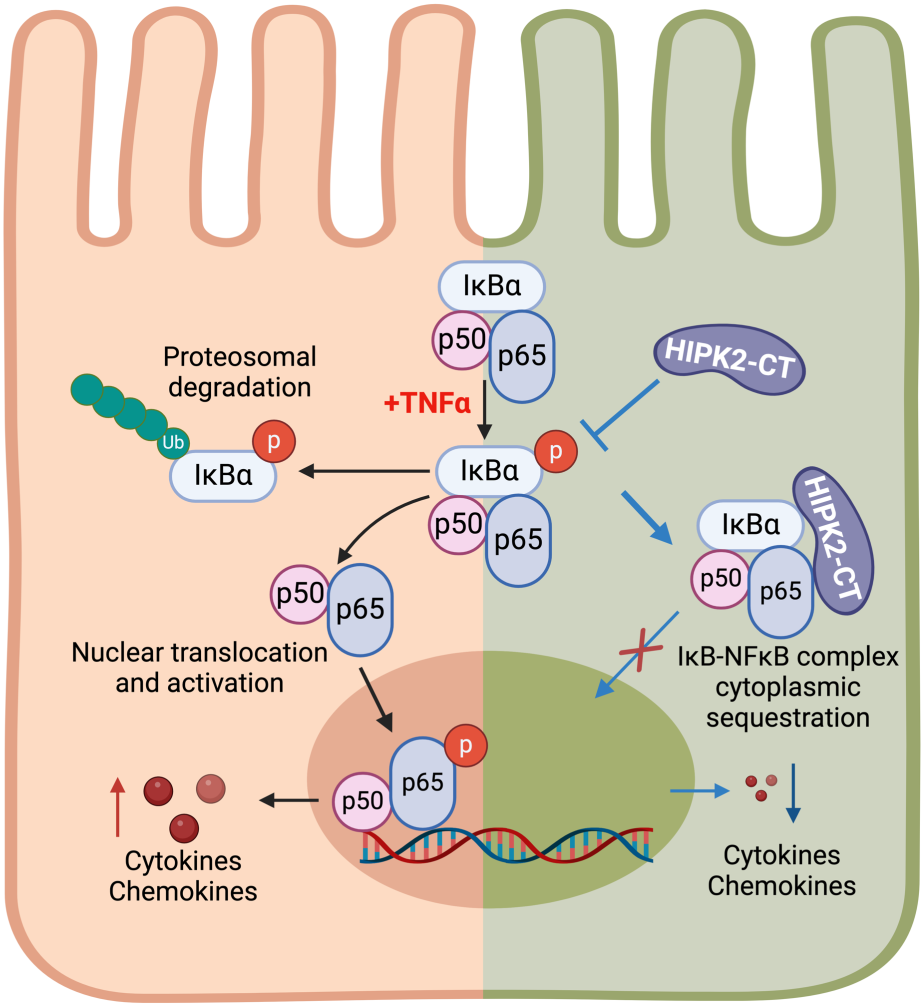

Supp. Figure 4: Schematics of HIPK2 C-terminal domain function in dampening inflammation in kidney tubular cells.

Supp. Methods

Expression constructs

All the expression constructs were generated using the PCR-based subcloning methods. The following primers were used to subclone the genes from cDNA library:

Human **Caspase-6**: F: 5'-AAA GCT TGC CAC CAT GAG CTC GGC CTC GGG GCT C-3' and R: 5'-TTG GTT TAA ACC GGT TAA TTA GAT TTT GGA AAG AAA TGC AGC-3'.

Human **active Caspase-6:** F: 5'-AAA GCT TAA GCC ACC ATG GAT GCA GCC TCC GTT TAC ACG CTG CCTG-3'; R: 5'-TTG GTT TAA ACC GGT TAA TTA GAT TTT GGA AAG AAA TGC AGC-3').

Human **FLAG-HIPK2-V5**, F:5'-TTG GCT AGC CAC CAT GGA CTA CAA GGA CGA CGA TGA CAA GCT TGC CCC CGT GTA CGA AGG TAT GGC CTC ACA TGT G-3' and R: 5'-AAC CGC GGC CGC TCT AGA TTC ATG TAG AAT CAA GTC CCA GAA GAG GAT TTG GAA TAG GTT TTC CTA TGT AAG GGT ACT GGT TGA CCT TGG-3'.

FLAG-HIPK2-CT: Using pcDNA4/Myc-His-B Mammalian Expression Vectors (Invitrogen, V86420), FLAG-tag was introduced into the vector at HindIII and BamHI sites by using a linker (F: 5'-AGC TCC CGG GCC ACC ATG GAC TAC AAG GAC GAC GAT GAC AAG CTT G-3' and R: 5'-GAT CCA AGC TTG TCA TCG TCG TCC TTG TAG TCC ATG GTG GCC CGG G-3'). HIPK2-CT was amplified by PCR with the following primers and subcloned into the modified vector (F: 5'-TAA AGC TTG GAT CCC TGG TGC CAG TCA ACA CCA GTC-3' and R: 5'-AAG GCG GCC GCG GTA CCT ATT ATA TGT AAG GGT ACT GGT TGA CCT TGG-3').

mCherry-p65: human-p65 was amplified by the primers (F: 5'- AAA GCT TGC TAG CCA CCA TGG ACG AAC TGT TCC CCC TCA TCT TC-3'; R: 5'- AAA GCT TAT TAA GCG CTG ATA TCG GAG CTG ATC TGA CTC AGC-3') and introduced into the mCherry plasmid purchased from Addgene (Cat#58476) with RE sites 'HindIII' and 'HpaI'

FLAG-HIPK2-CT-EGFP: cDNA fragment of human HIPK2-CT and EGFP were amplified with primers F: 5'-TAA AGC TTG GAT CCC TGG TGC CAG TCA ACA CCA GTC-3'; R: 5'- TTG GCG GCC GCA GCG CTT ATG TAA GGG TAC TGG TTG ACC TTG-3' and 5'- AGG CGG CCG CGA ATT CAC TAG TGA TTA GGG ATC CTA TTA CTT GTA CAG CTC GTC CATG-3', respectively. cDNAs were cloned and purified, introduced in the pcDNA4B-FLAG vector.

HIPK2 WT-EGFP-V5 construct, DNA fragment of EGFP with primers (F: 5'- TTG GCT AGC CAC CAT GGT GAG CAA GGG CGA GGA GCT G-3'; R: 5'- ATG GCG GCC GCA AGC TTG TAC AGC TCG TCC ATG CCG AGA GTG ATC C-3') were amplified and introduced into previous FLAG-HIPK2-WT-V5 construct.

Primers used for qPCR

Gene	Primer sequence (forward)	Primer sequence (reverse)
Ms-Gapdh	GCCATCAACGACCCCTTCAT	ATGATGACCCTTTTGGCTCC
Ms-Ccl2	CCAGCAGCAGGTGTCCCAAAGA	TTCTTGGGGTCAGCACAGACCTCTC
Ms-Il-6	ACTTCACAAGTCGGAGGCT	TGCAAGTGCATCATCGTTG
Ms-Tnfa	GACGTGGAACTGGCAGAAGAG	TTGGTGGTTTGTGAGTGTGAG
