# Supplementary figures and images for: HIPK2 C-terminal domain inhibits NF-κB signaling and renal inflammation in kidney injury
Source: JCI Insight. 2024 Mar 21;9(8):e175153. doi: 10.1172/jci.insight.175153 (PMC11141872; doi:10.1172/jci.insight.175153)

Uncropped WB images

Fig. 1C

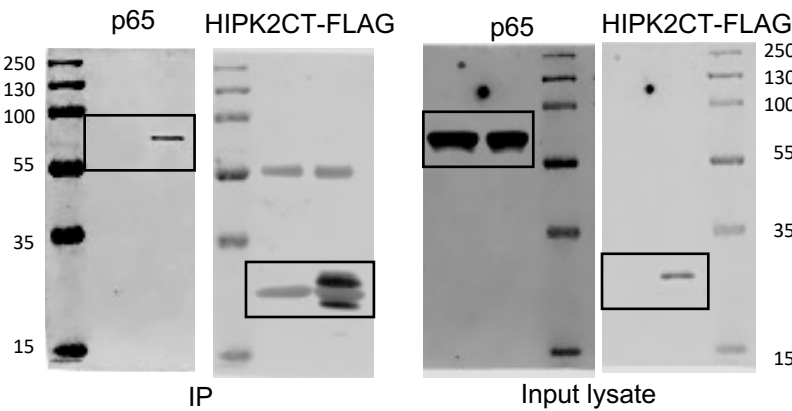

Fig. 1D

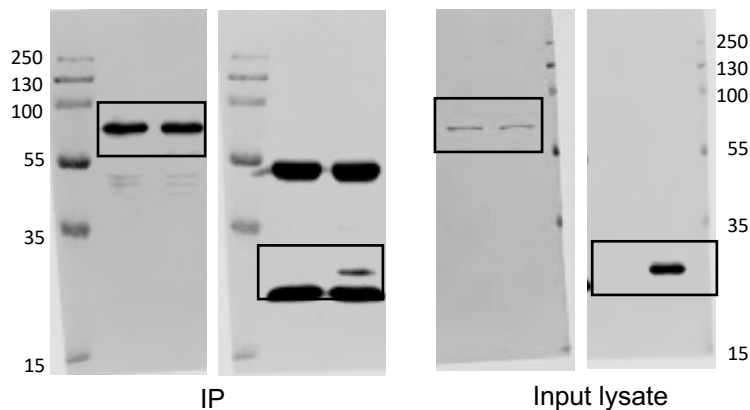

Fig. 2A

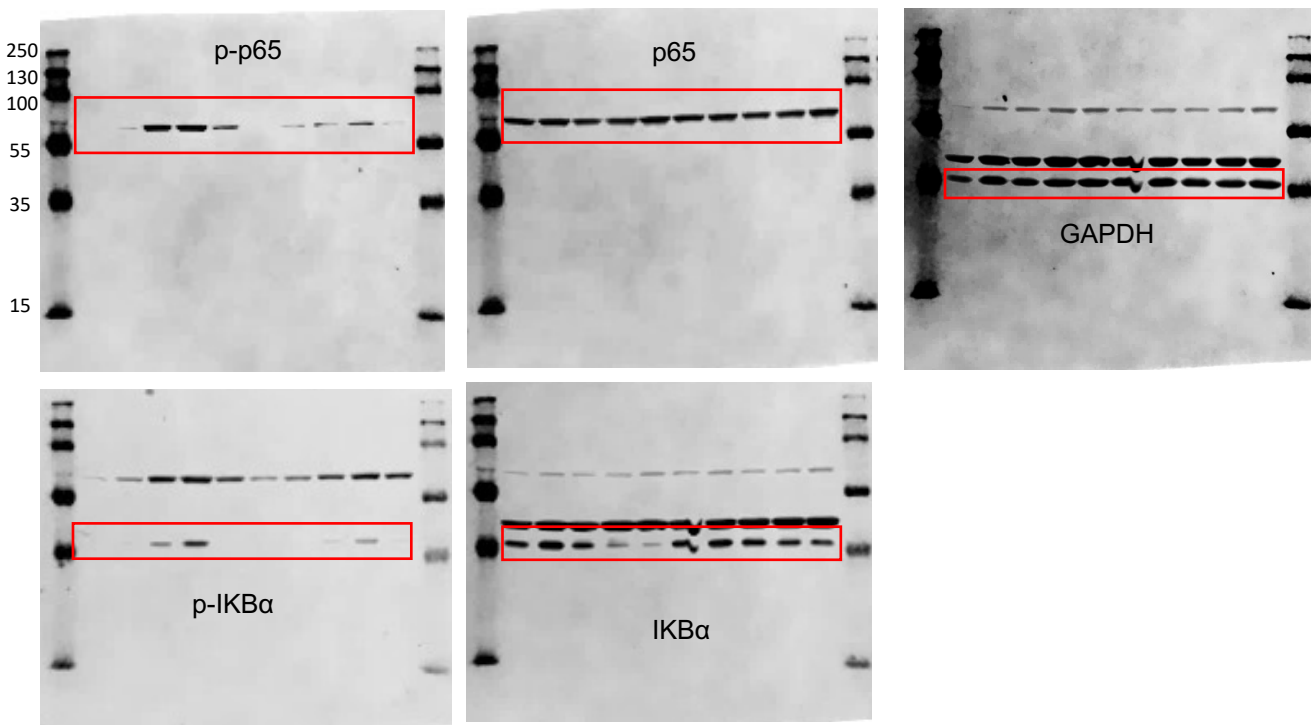

**Fig. 2C**

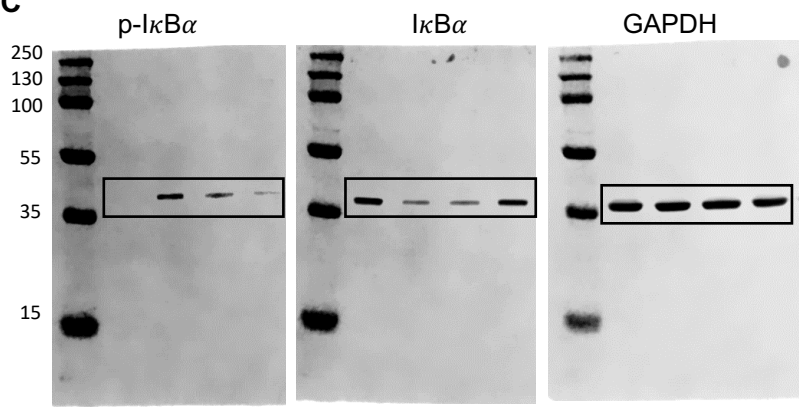

**Fig. 2D**

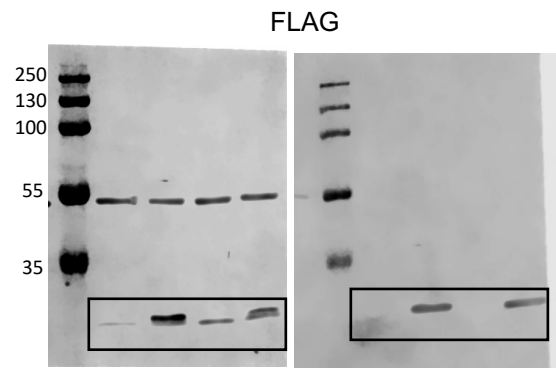

**Fig. 4B**

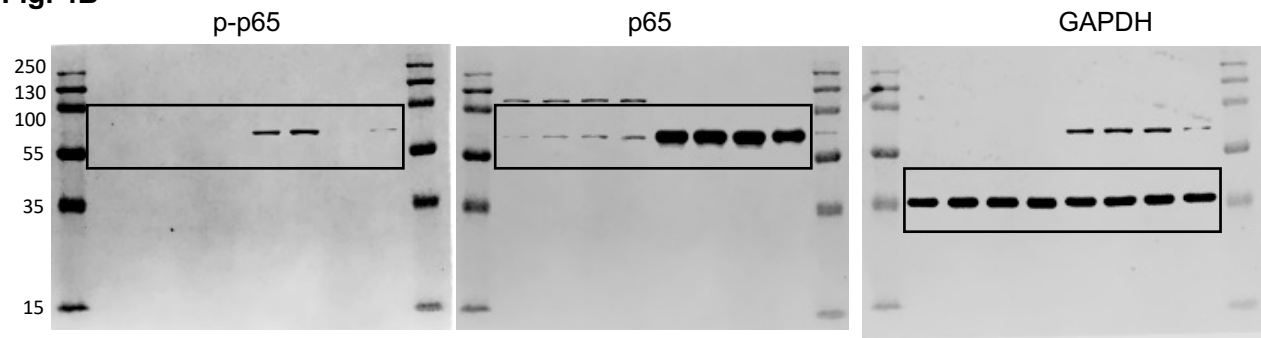

**S. Fig. 1B**

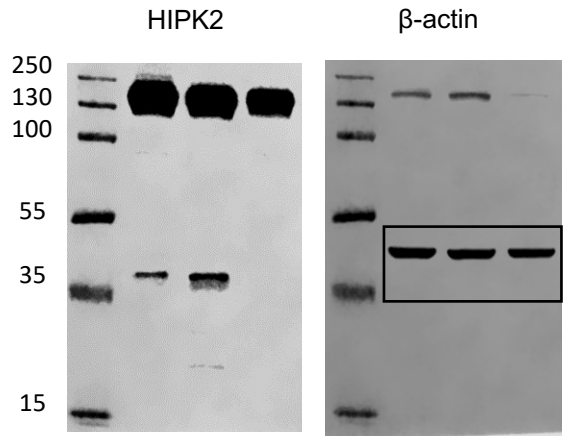

Supplement: Unedited blot and gel images [file jciinsight-9-175153-s186.pdf]
